# Long-term pain and health economic outcomes in adults receiving multidisciplinary CBT for chronic pain: the role of psychological inflexibility

**DOI:** 10.3389/fpain.2025.1547540

**Published:** 2025-04-28

**Authors:** Sophia Åkerblom, Lance M. McCracken, Marcelo Rivano Fischer, Sean Perrin

**Affiliations:** ^1^ Department of Pain Rehabilitation, Skåne University Hospital, Lund, Sweden; ^2^ Department of Health Sciences, Lund University, Lund, Sweden; ^3^ Department of Psychology, Uppsala University, Uppsala, Sweden; ^4^ Department of Psychology, Lund University, Lund, Sweden

**Keywords:** chronic pain, long-term outcomes, health economic outcomes, psychological inflexibility, mediator, predictor

## Abstract

**Background:**

Little is known about whether the recommended, non-pharmacological treatments for chronic pain yield reductions in healthcare utilization, social costs and increased productivity in actual practice.

**Methods:**

The primary aim of this study (*n* = 232) was to conduct secondary analyses of health economic outcomes using data from national registries combined with clinical outcome data from a large pain center in Sweden conducting multidisciplinary treatment based on a cognitive behavioral approach. Specifically, pain-related and health economic outcomes at post-treatment and one, two and three years after discharge were examined. In an exploratory fashion, we also investigated whether sociodemographic characteristics, pain-related variables, and psychological *in*flexibility predicted these long-term pain-related and health economic outcomes. We also examined psychological *in*flexibility as a potential mediator of these outcomes.

**Results:**

Small and moderate sized improvements in pain, pain interference, and depression observed at post-treatment were mostly maintained at both the 1- and 3-year follow-up. A very similar pattern was observed for health economic outcomes, with 1-year follow-up gains being maintained at long-term follow-up. Baseline psychological *in*flexibility predicted long-term pain-related outcomes, but not health economic outcomes. Changes in psychological *in*flexibility during treatment and follow-up mediated long-term pain-related outcomes and the total number of health care visits.

**Conclusions:**

The present findings add to a small body of literature indicating that the improvements in pain and related difficulties following multidisciplinary, pain-focused, CBT programs persist at least three years following treatment, and these are accompanied by modest improvements in health economic outcomes over the same interval. Psychological *in*flexibility seems to be predominately associated with long-term clinical outcomes in pain management, and it also appears relevant to the number of health care visits.

## Introduction

Approximately twenty percent of adults in Europe, including Sweden, suffer from chronic pain of moderate to severe intensity with significant economic impacts for the individual and society (1). A pan-European study found 61% of respondents with persistent pain were unable or less able to work, 32% had changed or lost their jobs, and 60% had visited their doctor 2–9 times during the last six months because of pain (1). A national registry study of 840,000 patients in Sweden estimated the yearly cost of chronic pain at €32 billion (2). Similarly high estimates have been found in other European countries (1). Less is known about whether chronic pain treatments lead to reduced healthcare utilization and social costs and increased productivity (3, 4).

The first-line recommended treatment for chronic pain in Sweden (5) and elsewhere (6) is multidisciplinary treatment including pain-focused cognitive behavioral therapy (CBT). This treatment yields small to moderate effects for pain severity, pain interference, health-related quality of life, disability, distress, and mood when compared with treatment as usual/waiting list (3, 4). However, outcomes are typically assessed at post-treatment with relatively short-term follow-ups of 12 months or less. Also, there are complementary outcomes that only rarely appear in the literature, such as health care utilization, work ability, return to work, and medication use (3, 7).

Systematic reviews of the economic benefits of non-pharmacological treatments for chronic pain identify a relatively small number of studies, heterogeneous in nature, and often reliant upon patient self-reports to estimate health economic outcomes (8, 9). For individuals with chronic pain in Sweden, two exceptions are registry studies of patients undergoing interdisciplinary pain rehabilitation—each of these find reductions in social insurance compensation, one and two years after discharge (10, 11).

Psychological flexibility is defined as the capacity to act with openness, awareness, and in line with one's chosen values while experiencing unwanted thoughts, emotions, or bodily symptoms (12). The psychological flexibility model is both a model of behavior of people suffering from chronic pain or other difficulties, and also a broadly applicable model of human behavior and wellbeing in general. The model includes emotional, cognitive, attentional, self-related, motivational, overt behavioral flexibility processes that together entail effective performance as well as a parallel set of inflexibility processes that entail ineffective performance and poor health (12). In previous studies, we have shown that processes from the psychological flexibility model as applied to chronic pain (12, 13), particularly psychological *in*flexibility, act as a predictor and mediator of pain-related outcomes for multidisciplinary pain-focused CBT delivered in a specialist pain clinic in Sweden (14, 15). We have not previously examined health economic outcomes from this same treatment center nor the role played by psychological *in*flexibility in these outcomes.

The purpose of this study was to conduct secondary analyses of data from a large multidisciplinary pain center in Sweden, combined with data from national registries, to focus on pain-related and health economic outcomes at post-treatment and one, two and three years after discharge. In an exploratory fashion, we investigated whether sociodemographic characteristics, pain-related variables, and psychological *in*flexibility, predicted these long-term pain-related and health economic outcomes. We also examined psychological *in*flexibility as a potential mediator of these outcomes.

## Methods

### Participants and procedure

Participants in this study (*n* = 232) were consecutive referrals undergoing treatment at the Pain Rehabilitation Unit at Skåne University Hospital, in southern Sweden, between February 2014 and December 2015. This is tertiary, government supported, regional, center for chronic pain and related disability. The sociodemographic variables reported as a part of this study (age, gender, country of birth, level of education) were assessed at baseline (pre-treatment). The clinical variables were assessed at pre-treatment, post-treatment, and 1 and 3 years after discharge. The 3-year-follow up was specifically undertaken for this study. Participants were mailed questionnaires 1 year and 3 years after discharge from the day treatment program (the follow-up assessments). Health economic variables were obtained from national registries for all participants before treatment participation (pre-treatment) and at 1, 2, and 3 years after treatment at the clinic. All participants provided written informed consent. The study was approved by the Regional Ethical Review Board in Lund, Sweden (2013/381). It is in line with the Strengthening the Reporting of Observational studies in Epidemiology guidelines (STROBE) and includes the necessary items to properly report an observational study according to the STROBE checklist (von Elm et al., 2007).

### Treatment program

The treatment was multidisciplinary, outpatient, and based on a cognitive behavioral approach, delivered by teams with training in CBT and extensive knowledge of pain rehabilitation. An individual rehabilitation plan was formulated during individual appointments and then followed for each patient. The treatment was biopsychosocial in orientation and primarily group-based with the focus to help patients to develop more adaptive ways of thinking and behaving in relation to pain. The emphasis was on improving practical skills, knowledge, and awareness with methods including physical exercises and relaxation (physiotherapist); pain and medication (physician); education in work-related and national insurance issues (social worker); ergonomics, time-use adaptations, problem-solving strategies, and everyday occupational performance (occupational therapist); and thoughts, emotions, behaviors, communication, and goal-setting methods (clinical psychologist). The treatment was based within a broad CBT framework and not specifically on the psychological flexibility model of chronic pain. The program was delivered over five weeks (18 active treatment days 5–7 h per day) followed by a two-month “homework” phase, where patients work on personalized goals with support from the multidisciplinary team.

### Measures

#### Depression

Depression was measured using the seven depression items from the 14-item Hospital Anxiety and Depression Scale (HADS) (16). Items are rated on a 0–3 severity scale, yielding total scores for anxiety and depression; higher scores correspond to higher levels of depression/anxiety over the past week. The cut-off score for the depression subscale is: 0–7 for non-cases; 8–10 for doubtful cases; and 11–21 for clinical cases (16). Consistent with the original, the Swedish version used in the current study has satisfactory internal reliability for the total (*α* = .90) and depression scales (*α* = .82) (16, 17).

#### Psychological inflexibility

Psychological *in*flexibility was assessed with the 12-item Psychological Inflexibility in Pain Scale (PIPS) (18); 8 items assess pain avoidance and 4 items assess cognitive fusion. Items are rated on a 7-point scale (*1* *=* *never true; 7* *=* *always true)* with higher scores corresponding to greater psychological *in*flexibility. The Swedish original (used in this study) has acceptable validity and reliability [*α* = .89 (avoidance), .66 (fusion) and .87 (total scale)] (18).

#### Pain intensity

Pain intensity was captured with the single-item Numerical Rating Scale (NRS) where the respondent rates their pain intensity during the past week on an 11-point scale (0 = *no pain*; 10 = *worst possible pain*). The NRS is widely considered to be a valid measure of pain intensity and sensitive to the effect of pain-focused treatment (19, 20).

#### Pain interference

Pain interference was assessed with the 11-item pain interference subscale from the Multidimensional Pain Inventory, Version 2 (MPI-2) (21). Respondents use a 7-point scale (0 = *never*; 6 = *very often)* to rate how pain interferes in family and marital functioning, work and work-related activities, and social and leisure activities. Higher scores indicate greater functional impairment from pain. The original and Swedish version used in this study have acceptable psychometric properties (*α* = .72-.90) and is sensitive to the effects of pain-focused treatments (14, 22).

#### Health economic variables

Four health economic variables were obtained from Swedish national registries for the 232 participants: (1) number of all health care visits over the past year; (2) cost of all health care visits over the past year; (3) number of compensated sick days over the past year; and (4) cost of compensated sick days over the past year. The cost variables reflect the total cost over the past year in Swedish Kronor (SEK). At the time the cost variables were obtained from the health care registries, between 10 and 11.2 SEK purchased one Euro. Regarding compensated sick days, this includes gross days and costs of sickness cash benefits, work injury sickness cash benefits, rehabilitation cash benefits, and preventive sickness cash benefits. Again, health economic variables were obtained from national registries for all participants before treatment participation (pre-treatment), and at 1, 2, and 3 years after treatment at the clinic. The values at each assessment represents the mean for the preceding year.

### Statistical analyses

Descriptive statistics for the outcome measures were produced. Effect sizes (pre-to-post-treatment and pre-to-follow-up) were calculated for the mediator and outcome variables and interpreted (*Cohen's d*) as small (≥.2), medium (≥.5), and large (≥.8) (23).

#### Multiple predictor analyses

We conducted separate multiple linear regressions for each outcome. In the regressions, we explored whether pre-treatment values for sex, age, education-level (12 years education or less vs. more than 12 years education), pain duration, and total score on the measure of psychological *in*flexibility in relation to pain predicted treatment outcomes as indexed separately by total scores on the depression, pain interference, pain intensity measures and the number of heath care visits, costs of health care visits, number of compensated sick days, and costs of compensated sick days– all at the 3-year follow-up. The baseline value on the outcome measure under investigation was included together with the other independent variables to control for pre-treatment variation in the outcome variables.

#### Mediator analysis

In the mediator analysis we strictly focused on psychological *in*flexibility as a potential mediator. Mediation is the effect of the independent variable (X) on the dependent variable (Y) through another clinical variable (M). The cross-product a*b provides an inference as to whether and to what degree M is functioning as a mediator of the effect of X on Y, with *a* representing the relation of X to M, and b representing the relation of M to Y adjusted for X (24). Mediation studies without reference to controls and using data from repeated measurements of the same individuals are relatively common (25). In such cases, change scores on the mediators (M) and outcome variables (Y) are assumed to be influenced by the treatment program and X represents the effect of time (pre-treatment to 3-year follow-up). Even if the empirical evidence from this method is not as conclusive as mediation analyses using control groups, and random assignment, it can contribute with increased insight of change processes during treatment (26). In the current study, we assessed whether changes from pre-treatment to 3-year follow-up on the measures of pain intensity, pain interference, and depression, number of heath care visits, cost of health care visits, number of compensated sick days, and cost of compensated sick days, all used as a proxy for treatment effects, were mediated by changes in in psychological *in*flexibility from pre-treatment to 3-year follow-up. Investigation of changes during the entire study period (pre-treatment to 3-year follow-up) allows us to determine whether change in psychological *in*flexibility might contribute to change in outcomes during this time. The bootstrapping method with bias-corrected confidence estimates was used to test the significance of the indirect effect (27). The significance of the indirect or mediating effect is directly measured by the cross-product a*b. Confidence intervals are derived from an obtained distribution of a*b scores and if lower and upper bounds do not contain zero, the indirect effect is significant at the level specified in the analysis. All analyses were done using SPSS (Version 29) and the mediation analyses were conducted with 5,000 bootstrapped samples, with the algorithms and syntax for SPSS accessible online (25).

Incomplete data was handled using recommended procedures (28, 29). Little's MCAR test (Little, 1988) suggested that the data were missing completely at random, Hence, missing values were imputed at the item level using the Expectation-Maximization method (EM), while all available data were used if data were missing at the variable level (30, 31).

## Results

### Descriptive analyses

Table 1 presents the means, standard deviations, and effect sizes (Cohen's *d*), for the clinical variables at each assessment for the 232 study participants. Not reported in Table 1; the most frequently reported pain diagnoses were fibromyalgia (40.5%); neck-related pain (19.4%); and low back pain (4.7%); and approximately half (49.5%) of the participants had a pain duration of more than five years. A minority of the sample was male (14.2%) and the mean age was 41.6 (SD: 9.9). More than half of the participants had 12 years of education or less (63.2%).

**Table 1:** Means and standard deviations for the clinical and mediator variables at each assessment and effect sizes for each variable.

Variable	Pre-treatment M (SD)	Post-treatment M (SD)	1-year follow-up M (SD)	3-year follow-up M (SD)	Pre-treatment to post-treatment Cohen's *d* (*n* = 217–210)	Pre-treatment to 1-year follow-up Cohen's *d* (*n* = 164–156)	Pre-treatment to 3-year follow-up Cohen's *d* (*n* = 145-141)
Pain intensity	7.27 (1.40)	6.58 (1.80)	6.47 (1.62)	6.50 (1.90)	.39	.46	.33
Pain interference	4.69 (.80)	4.17 (.99)	4.07 (1.21)	3.89 (1.26)	.59	.59	.60
Depression	9.88 (4.26)	7.11 (4.14)	7.88 (4.66)	7.84 (4.50)	.63	.46	.39
Psychological *in*flexibility	60.32 (11.93)	50.77 (12.51)	48.97 (14.13)	49.60 (16.44)	.87	.88	.59

Pain intensity was assessed with the Numerical Rating Scale, pain interference with the Multidimensional Pain Inventory, depression with the Hospital Anxiety and Depression Scale, psychological *in*flexibility with the Psychological Inflexibility in Pain Scale.

Table 2 presents the means and standard deviations for the health economic outcome variables at all time points, and effect sizes (Cohen's *d*), using all available data for the 232 participants in the study. Again, the values at each assessment represents the mean for the preceding year.

**Table 2:** Means and standard deviations for the health economic variables at each assessment and effect sizes for each variable.

Variable	Pre-treatment M (SD)	1-year follow-up M (SD)	2-year follow-up M (SD)	3-year follow-up M (SD)	Pre-treatment to 1-year follow-up Cohen's *d* (*n* = 188-168)	Pre-treatment to 2-year follow-up Cohen's *d (n* = 188-168	Pre-treatment to 3-year follow-up Cohen's *d* (*n* = 188-157)
Total number of health care visits	44.95 (27.97)	31.84 (22.13)	28.58 (22.77)	30.03 (24.29)	.55	.59	.51
Total cost of health care visits (SEK)	57,015.78 (51,723.89)	29,653.84 (41,045.12)	31,383.99 (52,309.49)	23,202.35 (43,195.29)	.65	.50	.70
Total number of compensated sick days	222.02 (148.04)	247.70 (143.10)	181.27 (158.15)	141.39 (158.14)	-.15	.20	.37
Total cost of compensated sick days (SEK)	96,219.30 (75,344.77)	105,511.48 (75,384.48)	76,662.02 (77,071.71)	58,687.30 (74,167.15)	-.12	.20	.36

SEK, Swedish Kronor (10-11.2 SEK = 1 Euro, approximately); Values for the health economic variables are the means for the previous year.

Computed effect sizes for the clinical outcomes and mediator (psychological *in*flexibility) ranged from small to large, with nine out of twelve values falling in the medium range or larger. As shown in Table 1, the smallest effects were for pain intensity and the largest for psychological *in*flexibility. The effect sizes for the health economic outcomes in Table 2 ranged from small to medium, although the sick days data, for both number of days and costs, at one year showed a reverse of the expected effects in that the number of days and costs increased. Total health care visits and total health care costs both showed consistent medium effects at each follow-up. While the sick days data, again both number of days and costs, improved at the two-year and three-years marks, the effects were small.

### Multiple predictor analyses

Two significant findings emerged in the regressions. Participants who reported higher psychological *in*flexibility at baseline reported significantly worse depression at the 3-year follow-up (*β* = 0.195, *t* = 2.166, *p* = .032). Participants with 12 years education or less reported a higher number of compensated sick days at 3-year follow-up (*β* = .165, *t* = 2.032, *p* = .044).

### Mediator analysis

Table 3 presents the mediation results. Psychological *in*flexibility mediated changes for all pain-related outcomes (depression, pain intensity, pain interference) and mediated the total number of health care visits. No other mediation effects were identified.

**Table 3:** Results of mediation analysis.

Dependent Variable	*N*	Mediator	Results for indirect effects (a*b)	
			Point-Estimate (SE)	95% CI LL, UL
Pain intensity	141	Psychological *in*flexibility	.212 (.114)	.006, .461
Depression	144	Psychological *in*flexibility	1.051 (.302)	.516, 1.689
Pain interference	145	Psychological *in*flexibility	.301 (.073)	.172, .457
Total number of health care visits	142	Psychological *in*flexibility	3.963 (1.842)	.842, 8.091
Total cost of health care visits (SEK)	116	Psychological *in*flexibility	452.707 (3,854.575)	−6,786.016, 8,654.477
Total number of compensated sick days	124	Psychological *in*flexibility	16.598 (12.009)	−6.546, 41.309
Total cost of compensated sick days (SEK)	124	Psychological *in*flexibility	8,199.991 (5,855.827)	−2,810.776, 20,627.030

LL, lower limit; UL, upper limit.

The indirect effect is statistically significant if the confidence interval (CI) does not include zero.

## Discussion

Chronic pain can be a highly disabling and costly problem for the affected individuals and society. While moderately effective treatments in the form of pain-focused CBT programs are available, little is known about their impact on broader health economic outcomes in actual practice, or the processes through which they yield clinical and health economic improvements. This study aimed to address this literature gap by conducting secondary analyses of data from a large multidisciplinary pain center in Sweden, combined with data from national registries, to focus on pain-related and health economic outcomes at post-treatment and one, two and three years after discharge. In addition, we investigated whether sociodemographic characteristics, pain-related variables, and psychological *in*flexibility, predicted these long-term pain-related and health economic outcomes. We also examined psychological *in*flexibility as a potential mediator of these outcomes. To our knowledge, this is the first study to evaluate whether psychological *in*flexibility, a variable that has been shown to predict and mediate one-year pain outcomes in multidisciplinary, pain-focused CBT programs, predicts or mediates both changes in clinical and health economic outcomes and whether it does so over the longer term.

Our findings add to a limited literature on the durability of pain-related outcomes more than 12 months after completion of a multidisciplinary, pain-focused treatment program. In the present study, the small and moderate sized improvements in pain, pain interference, and depression observed at post-treatment were mostly maintained at both the 1- and 3-year follow-up.

A very similar pattern was observed for the health economic outcomes, with gains attained by 1-year follow-up being maintained at long-term follow-up. The largest improvements were found for the total number and costs of healthcare visits, with moderate effect sizes at the 1-year follow-up and remaining stable at both the 2- and 3-year follow-ups. The costs of healthcare visits declined by 48% during the first year after treatment and at the time of the 3-year follow-up by 59% relative to the year up to commencement of treatment. The gains in respect of days with and costs of sick pay were much more modest. There was a slight worsening in this variable at the 1-year follow-up, with small gains not observed until the 2- and 3-year follow-ups. While the decline in the number of sick days and associated costs was in the small range, this nonetheless represented a 39% reduction in the cost attributable to compensated sick days by the time of the 3-year follow-up. While further studies are needed, small reductions in sick day costs following pain-focused treatment are potentially important given the high prevalence of chronic pain in the general population.

The mean number of health care visits, even after the treatment period, was high for this treatment sample. We note that this tertiary care population in Sweden can be considered to have a high health care utilization (32). However, this study was not able to determine whether all these contacts were due to pain and whether a small subgroup with a very high health care utilization pattern was driving these results or if the utilization was more evenly divided throughout the sample. Future studies need to investigate trajectories of healthcare utilization in different subgroups with chronic pain.

In an exploratory fashion, we sought to examine potential predictors of these pain-related and health economic outcomes. In respect of predictors, individuals with 12 years of education or less reported a higher number of compensated sick days at the 3-year follow-up. This finding warrants further investigation. Scores on a measure of psychological *in*flexibility at baseline did not predict the health economic outcomes. However, psychological *in*flexibility did predict greater depression at the 3-year follow-up. The latter is consistent with previous findings from our research group which identified psychological *in*flexibility as a predictor of higher scores for depression and pain interference at 12-months follow-up on patients from this clinic (15). These findings suggest that individuals with high levels of psychological *in*flexibility at baseline experienced worse treatment outcomes. This is understandable as psychological *in*flexibility can be regarded as a lack of basic skills for seizing opportunities and making the best of it. Participants high in psychological *in*flexibility might benefit from more intensive targeting of this process during treatment. One way to improve the individualization, and efficacy, of group-based CBT programs for chronic pain could be the administration of a self-report measure of psychological *in*flexibility during the assessment phase. Patients scoring high on this measure could be offered one-to-one or small group interventions specifically aimed to increase their levels of psychological flexibility in addition to the standard group treatment. Another way to address this problem could be individual and personalized process-based therapy (PBT), where evidence-based processes of change, such as psychological *in*flexibility, are addressed based on idiographic assessment, to target the needs and reach the goals of each individual (33).

As noted in the introduction, we have previously found that changes during treatment in different facets of the psychological flexibility model as applied to pain, including psychological *in*flexibility, mediated pain intensity, pain interference and depression; all primary targets of pain-focused treatments (14, 15). These findings were partly replicated in this long-term follow-up study as changes in psychological *in*flexibility between pre-treatment and the 3-year follow-up mediated changes in pain interference, depression, and pain intensity over the same interval. In addition, changes in psychological *in*flexibility over this 3-year interval mediated the total number of health care visits between pre-treatment and the long-term follow-up. These findings warrant further investigation in larger samples allowing more complex modelling strategies. It is possible that changes in psychological *in*flexibility, pain, and associated emotional difficulties, interact with other patient characteristics, symptoms, or circumstances (e.g., age, multimorbidity, and relationship status/support) to impact healthcare usage and thus costs. Overall, the present findings suggests that reductions in psychological *in*flexibility partly underpins the longer-term gains observed following treatment in a multidisciplinary CBT program for chronic pain, including small reductions in the number of health care visits. Again, one way to potentially improve outcomes for adults seeking treatment for chronic pain further could be more detailed and precise assessment and targeting of psychological *in*flexibility. Psychological flexibility has been pointed out as a counterweight to many forms of psychopathology, an essential part of psychological functioning, and a fundamental aspect of health (34–36), and its broad relevance to the investigated outcomes lends support to such notions.

The present findings must be viewed within the context of certain limitations. While the sample size was relatively large (*n* = 232), there was no un-treated or non-pain comparison group, and the data were obtained from a single, regional pain clinic. The last follow-up in this study occurred 36 months after treatment completion, which compares favourably to the 3- to 12-month follow-ups usually reported for pain-related outcomes in the literature. However, given the chronicity of pain in this sample, and as a condition more broadly, even longer-term follow-ups are needed, and again with appropriate comparisons groups. The health economic data reported in this study were not based on self-report, as in most studies, and instead obtained from Swedish government registries. Nevertheless, these findings may not generalize across pain clinics in Sweden or to pain services outside the country. The generalizability to other clinical settings might also be limited by the fact that the sample included a large proportion of women and participants diagnosed with fibromyalgia. This study included a large number of predictor variables and comparisons, which may increase the risk of type I errors. Also, this study did not control for additional treatment received during the years following completion of the pain program. Finally, future studies need to evaluate a broader range of health economic outcomes, including both direct and indirect costs, when considering the impact of pain-focused CBT.

In conclusion, the present findings add to a small body of literature indicating that the improvements in pain and related difficulties following multidisciplinary, pain-focused CBT programs appear to persist at least three years following treatment. Small to moderate improvements in health economic outcomes also occur during this same period. The present results add to previous research demonstrating that a patient's level of psychological *in*flexibility is associated with clinical outcomes in pain management. The findings expand on the literature by suggesting that changes in psychological *in*flexibility during multidisciplinary, pain-focused CBT appear important for achieving improved clinical outcomes over the longer term, and relevant to long-term health economic outcomes, at least with respect to the number of health care visits.

## Data Availability

The datasets presented in this article are not readily available due to ethical restrictions. Requests to access the datasets should be directed to sophia.akerblom@med.lu.se.
